# Editorial

**Published:** 1871-07

**Authors:** 


### Editorial.

Graded Classes and Consecutive Study in the Medical Colleges.—On another page will be found a notice of an important change in the plan of instruction in the Medical Department of Harvard University, at Boston. It is the leading medical school in the New England States, and its adoption of the graded class system will have much influence in furthering a very desirable change in the medical colleges generally.

We are glad to see that most of the medical journals notice and commend the movement in the Boston School. Some of them, however, accompany their notices with very singular comments. For instance, the editor of the *New York Medical Record,* in his June number, alluding to the division of the curriculum and the gradation of the classes, says: "It strikes us that the promises for the success of such a feature in college instruction are sufficiently good to invite a fair trial. *The initiative has, in fact, been already taken. The Woman's Medical College of this city, as we are at present informed, has the credit of being the pioneer in such a movement. For a year* or more, it has so arranged the plan of instruction as to secure a gradation of studies through three years of the student's course. The class in attendance is divided into three grades, corresponding respectively to the three years of study. An examination is held at the end of each term for promotion."—(See *N. Y. Medical Record,* p. 158).—We have many times been reminded that a large part of the people in the Atlantic cities have no knowledge of any country or people west of the Alleghany Mountains. We did not suspect, however, that the intelligent editor of the *Medical Record* belonged to that class, until we read the editorial from which the above paragraph is quoted.

But his representation of the Woman's Medical College in New York as the *pioneer* institution adopting, and the republication of his own advocacy of the policy five years since as evidence of his early sanction of, the plan, clearly shows that if he is not entirely ignorant of the fact that there is an inhabited country west of the Alleghany range, and a little town of 300,000 located near the south end of a small pond known as Lake Michigan; he is at least oblivious to the fact, that so early as the fall of 1859, a Medical College was organized and put into active operation in the city of Chicago, for the express purpose of establishing the system of medical college instruction on a broader and more systematic basis.

Said college adopted at the beginning a full curriculum of branches, divided it into two groups, and the students into two classes—called junior and senior—with full examinations at the end of each term. It also adopted a regular college term of five months, with a supplementary summer clinical term of four months.

After a few years, when the success of the undertaking had been fully established by a steadily increasing patronage, it adopted, in 1868, the full division of its curriculum into three series of branches, and its students into three classes—junior, middle, and senior—corresponding to the three years of study; extended its regular lecture term to five and a-half months, with a supplementary summer clinical term of three months; and has so continued to the present time. Two years since, a new medical school was organized in St. Louis—another little town, on the river known to minute geographers as the Mississippi—which fully adopted the system of graded classes and consecutive teaching. For further information on the subject, we would refer our respected Eastern co-temporary to the Annual Announcement of the Chicago Medical College, and a book published some fifteen or twenty years since, entitled, "History of Medical Education and Institutions in the United States."

---

IMPOSITION.—Some scalawag, calling himself "Dr. H. F. Adams," appears to be perambulating about in Kansas, distributing handbills containing the following certificate, which is a barefaced forgery from beginning to end:

"We, the undersigned, physicians, of the State of Illinois, who are personally acquainted with DR. H. F. ADAMS, of Chicago, Ill., can heartily and cheerfully recommend him as one of the most bold and successful surgical operators in the northwest. And we also know him to be a regular GRADUATE of Medicine and Surgery, of the College of Physicians and Surgeons, of New York City, as well as the University of Edinburgh, Scotland. The Doctor is worthy the public confidence generally.

Professor N. S. DAVIS, M.D., Chicago.
" ORRIN SMITH, M.D., "
" ADAMS ALLEN, M.D., "
J. B. GALLER, M.D., Warren, Illinois.
E. T. ALLEN, M.D., Alton, "
O. M. POND, M.D., Camp Point, Illinois."

We have received several copies from different parties. Some darkey should be hired to coat the fellow with tar, and give him a ride on a hemlock rail.

---

WISCONSIN STATE MEDICAL SOCIETY.—The Twenty-fifth Annual Session of the Wisconsin State Medical Society will be held in Bowman's Hall, in the city of Milwaukee, commencing on Wednesday, the 21st day of June prox., at seven o'clock P.M.

---

A NOTE OF PROGRESS.—The medical department of Harvard University at Boston, Mass., has become one of the largest medical schools in the country. We are gratified to find that its large and able faculty is not satisfied to settle down in the old beaten path of cramming all branches of medical science into the minds of first and third course students alike, but that it has adopted the system so long advocated by Dr. N. S. Davis, of Chicago, and adopted several years ago in the Chicago Medical College, of dividing the studies into three courses, one for each of the years required by custom in this country for attendance on medical lectures. The following is the curriculum of study adopted by the faculty:

*For the first year*—Anatomy, Physiology, and general Chemistry.

*For the second year*—Medical Chemistry, Materia Medica, Pathological Anatomy, Theory and Practice of Medicine, Clinical Medicine, Surgery and Clinical Surgery.

*For the third year*—Pathological Anatomy, Therapeutics, Obstetrics, Theory and Practice of Medicine, Clinical Medicine, Surgery and Clinical Surgery.

The advantages to the student of this plan of division of studies are self-evident, and it is to be hoped that other medical schools throughout the country will adopt the principle.

With the adoption of this principle in medical education must ultimately come the lengthening of the course of study. Three years do not give sufficient time for a thorough acquirement of even the theoretical part of medical knowledge, while much time should be devoted to witnessing and engaging in the practical application of this knowledge at the bedside of the sick, before the student enters upon the duties of a general practitioner of medicine.

This action of the medical department of Harvard University is the most hopeful sign of medical progress that has appeared for many years, and it should popularize this school with medical students.—*Philadelphia Medical and Surgical Reporter.*

CLINICAL SURGERY IN THE ROYAL GLASGOW INFIRMARY.—Dr. George Buchanan, in the year 1870, treated 499 cases of fracture and other surgical injuries, and of these 470 were dismissed, cured or relieved, and 29 died; giving a mortality of 5¼ per cent., which is a very small proportion, considering the nature of the injuries under treatment. They included 136 cases of fracture of various bones, both simple and compound, of which no less than 29 were of the thigh. Amongst the principal cases of operations were 9 cases of amputation of the thigh, 2 primary for injury, and 7 for disease—all successful. Such a result is most remarkable, and Dr. Buchanan states that he never met with the like success before. One lateral half of the tongue was excised in two cases, each of which made a good recovery. All the compound fractures were treated, as nearly as circumstances permitted, on the antiseptic principle, and there is no doubt that some were converted into simple fractures, with a celerity that could not have been achieved by any other plan. For treatment of compound fractures of the thigh and leg, Dr. Buchanan prefers the box splint, reaching from the buttock to beyond the foot, with foot-piece and hole for the heel. On the out or inside, as the wound is situated, is a slide splint fixed to a back one, or if there are wounds on both sides, the splint is made with a folding-door on each side, for changing the dressings. It is padded with well-carded oakum, which is antiseptic in itself—the fresh tarry smell being grateful to the patient, and the losseness of the material absorbs the discharge with great facility.—*The Doctor.*

---

ACUTE SYNOVITIS—INCISION INTO THE JOINT.—Mr. J. R. Jessop, F.R.C.S., in a lecture published by the *British Medical Journal*, states that he lately successfully followed Professor Lister's plan, and incised into the knee-joint of a patient aged twenty years, who suffered from acute synovitis, after the ordinary treatment in such cases had been tried, *i.e.*, rest, leeches, ice, evaporating lotions, salines, etc. Mr. Jessop made an opening into the joint, and in the axis of the thigh, commencing one inch above the patella, the opening was an inch long, but had to be enlarged to one and a-half inches to allow flakes of lymph to pass through, which were suspended in from eight to ten ounces of clear fluid. From the time the incision was made, the excruciating pain ceased, the fever disappeared, the swelling never returned, and the patient was sent from the Leeds Infirmary to a convalescent hospital, with a movable painless joint, within a month from the time of the operation.—*The Doctor.*

### Mortality for the Month of May, 1871.

| | |
|---|---|
| Accidents, burns | 2 |
| " brain compression | 3 |
| " crushed | 4 |
| " drowned | 10 |
| " fall | 5 |
| " run over by team | 2 |
| " steam railroad cars | 3 |
| " scalded | 1 |
| Aneurism of aorta | 1 |
| Apoplexy | 7 |
| Asphyxia | 1 |
| Atelectasis pulmonium | 1 |
| Births, premature | 11 |
| " still | 48 |
| Brain, compression of | 1 |
| " congestion of | 4 |
| " inflammation of | 9 |
| " softening of | 1 |
| Bronchitis | 12 |
| " capillary | 5 |
| Cachexia | 1 |
| Cancer of neck | 1 |
| " of stomach | 3 |
| " of uterus | 1 |
| Cerebral abscess | 1 |
| Chronic nervous prostration | 1 |
| Cholera infantum | 5 |
| " morbus | 1 |
| Consumption | 51 |
| Convulsions | 49 |
| " puerperal | 3 |
| " uræmic | 1 |
| Croup | 8 |
| " diphtheritic | 1 |
| " membraneous | 5 |
| Cyanosis | 2 |
| Cynanche maligna | 1 |
| " trachealis | 1 |
| Cystitis, chronic | 1 |
| Debility, general | 3 |
| Delirium tremens | 1 |
| Diphtheria | 10 |
| Diarrhœa | 3 |
| " chronic | 2 |
| Diarrhœa & complications | 3 |
| Dropsy, general | 5 |
| " of abdomen | 1 |
| " of chest | 1 |
| " & cirrhosis of liver | 1 |
| Dysentery | 7 |
| " acute | 1 |
| Empyema | 1 |
| Enteritis | 9 |
| Erysipelas | 3 |
| Fever, puerperal | 3 |
| " remittent | 1 |
| " and rupture of bloodvessel | 1 |
| " scarlet | 6 |
| " " & complicat's | 3 |
| " " malignant | 2 |
| " typhoid | 10 |
| Gangrene of arm | 1 |
| Gastritis | 4 |
| " and hypatitis | 1 |
| Gastro-enteritis | 1 |
| Hæmatemesis | 1 |
| Hemorrhage from nose | 2 |
| Heart disease | 7 |
| " dropsy of | 2 |
| " & liver disease of | 1 |
| " hypertrophy of | 3 |
| " rheumatism of | 2 |
| " valvular disease of | 7 |
| " clot, result of rheumatic fever | 1 |
| Hepatitis | 2 |
| " chronic | 1 |
| Hydrocephalus | 16 |
| Inanition | 13 |
| Kidneys, Bright's disease of | 1 |
| Laryngitis | 2 |
| Liver and stomach, obduration of | 1 |
| Lungs, congestion of | 6 |
| Manslaughter | 2 |
| Measles | 22 |
| " and complications | 19 |
| Meningitis | 6 |
| " cerebro-spinal | 5 |
| Metritis, puerperal | 1 |
| Myelitis | 1 |
| Old age | 11 |
| Paralysis | 3 |
| Peritonitis | 2 |
| " puerperal | 2 |
| Pneumonia | 36 |
| " broncho | 2 |
| " and complications | 3 |
| " typhoid | 2 |
| Pyæmia | 3 |
| Purpura | 1 |
| Rheumatism, acute | 3 |
| Scrofula | 1 |
| Septicæmia | 1 |
| Small-pox | 1 |
| Spine, injury of | 1 |
| Spina bifida | 1 |
| Stomach, hemorrhage of | 1 |
| Suicide by shooting | 3 |
| " by drowning | 1 |
| " by hanging | 1 |
| " by morphia | 1 |
| Tabes mesenterica | 11 |
| Teething | 1 |
| " and complications | 8 |
| Uterus, hemorrhage of | 2 |
| Whooping-cough | 3 |
| " and complications | 3 |
| Total | 525 |

### AGES.

| | |
|---|---|
| Under 1 | 156 |
| 1 to 2 | 62 |
| 2 to 3 | 29 |
| 3 to 4 | 20 |
| 4 to 5 | 17 |
| 5 to 10 | 27 |
| 10 to 20 | 33 |
| 20 to 30 | 40 |
| 30 to 40 | 42 |
| 40 to 50 | 30 |
| 50 to 60 | 25 |
| 60 to 70 | 23 |
| 70 to 80 | 10 |
| 80 to 90 | 8 |
| 90 to 100 | 1 |
| 100 to 101 | 1 |
| Unknown | 1 |
| Total, | 525 |

| | | | | | |
|---|---|---|---|---|---|
| Males, | 288 | Single, | 382 | White, | 517 |
| Females, | 237 | Married | 143 | Colored, | 8 |
| Total, | 525 | Total, | 525 | Total, | 525 |

COMPARISON.

Deaths in May, 1871,-- 525 | Deaths in May, 1870,--- 436 | Increase,---- 89
Deaths in April, 1871,------------ 423 | Increase,-------------------- 102

NATIVITY.

| | | | | | |
|---|---|---|---|---|---|
| Austria | 1 | England | 6 | New Brunswick | 0 |
| Bohemia | 8 | France | 0 | Norway | 9 |
| Canada | 5 | Germany | 70 | Nova Scotia | 1 |
| Chicago, Native | 66 | Holland | 0 | Switzerland | 0 |
| Chicago, Foreign | 212 | Ireland | 50 | Scotland, | 6 |
| U. S., other parts | 75 | Italy | 0 | Sweden | 10 |
| Denmark | 2 | New Foundland | 2 | Unknown | 2 |
| | | | | Total, | 525 |

MORTALITY BY WARDS FOR THE MONTH.

| Wards. | Mortality. | Pop. in 1870. | One death in |
|---|---|---|---|
| 1 | 8 | 6,531 | 816 |
| 2 | 14 | 14,338 | 1023 |
| 3 | 31 | 16,805 | 548 |
| 4 | 11 | 12,178 | 1107 |
| 5 | 14 | 11,605 | 829 |
| 6 | 30 | 19,486 | 649 |
| 7 | 27 | 13,849 | 513 |
| 8 | 53 | 22,994 | 442 |
| 9 | 47 | 27,278 | 580 |
| 10 | 19 | 13,750 | 724 |
| 11 | 22 | 14,988 | 681 |
| 12 | 17 | 13.976 | 887 |
| 13 | 7 | 8,943 | 1277 |
| 14 | 9 | 9,076 | 1007 |
| 15 | 52 | 20,382 | 390 |
| 16 | 20 | 13,975 | 698 |
| 17 | 25 | 17,118 | 685 |
| 18 | 26 | 17,069 | 683 |
| 19 | 9 | 8,738 | 971 |
| 20 | 9 | 13,628 | 1514 |

| Mortality. | |
|---|---|
| Accidents | 30 |
| Bridewell | 1 |
| County Hospital | 12 |
| Foundling Home | 4 |
| Home for Friendless | 1 |
| Hospital Alexian Brothers | 2 |
| Immigrants | 1 |
| Jewish Hospital | 1 |
| Marine Hospital | 1 |
| Mercy Hospital | 2 |
| Manslaughter | 2 |
| St. Luke's Hospital | 1 |
| St. Joseph Orphan Asylum | 10 |
| Scammon Hospital | 1 |
| Suicide | 6 |
| Total | 525 |

Mean Thermometer for month, 61°; Rain, 3,550 inches; Deaths daily, 16$\frac{4}{5}$.

It will be seen from the foregoing table that there is an increase by nearly all diseases, compared with the month of April, and that the infantile diseases incident to summer have already made their appearance. This arises from various causes, such as increase and change of temperature, greater rainfall, and difference in the direction and activity of the wind. In April, the prevailing wind was from the southwest; this month it was from the northeast. The effect of this change is observed in the fact that there is not much increase in the number of deaths in the wards lying in the north side and the Lake, while more than one-half (57) occurs in the 5th, 6th, 7th, and 8th wards, comprising the extreme southwestern part of the city, with a population of about one-sixth of the whole. No doubt the want of drainage, character of population, and the conditions surrounding them, had some influence, but not sufficient to cause such a great difference. Compared with the corresponding month of 1870, there is a decided increase, greater than the increase of population proportionately. In judging, however, of this fact, it is necessary to bear in mind that to May 1st, 184 less deaths occurred than for the same period in 1870, so that even with this increase we have had 83 less deaths than last year. There has, however, so far this year, been a less marked epidemic tendency than last. The month of May generally is one of the healthiest. In 1866 there were 275

deaths; 1867, 241; 1868, 321; 1869, 372; 1870, 436; and this year, 525. The mean temperature in 1866 was 58; 1867, 51; 1868, 55; 1869, 54; 1870, 65; and 1871, 61°. It will be seen that the mean daily temperature for the last two years has been unusually high, and with a corresponding increase in the number of deaths. While the mean daily temperature has not been as high as last year, the range of the thermometer has been 8° greater, with rainfall of nearly three inches more.

The general sanitary condition of the city was not as good as last year. This was owing to various causes, such as the increase of rain, making it impossible in certain localities to do anything, and the near approach to the completion of the deepening of the canal prevented the pumping of the Chicago River, lest it should interfere with the work, thus causing the offensive exhalations of the Chicago River to be longer continued than usual.

JOHN H. RAUCH, Sanitary Superintendent.

